# Supplementary material for: Enhancement of light emission in Bragg monolayer-thick quantum well structures
Source: Sci Rep. 2019 Jul 15;9:10162. doi: 10.1038/s41598-019-46646-2 (PMC6629672; doi:10.1038/s41598-019-46646-2)
Supplement: Supplementary file 1 — Supplementary information [file 41598_2019_46646_MOESM1_ESM.pdf]

Supplementary information for

Enhancement of light emission in Bragg monolayer-thick quantum well structures

Galia Pozina[1*], Konstantin A. Ivanov[2], Konstantin M. Morozov[3], Elizaveta I. Girshova[3], Anton Yu. Egorov[2,4], Stewart J. Clark[5], Mikhail A. Kaliteevski[2,3,4]

[1] *Department of Physics, Chemistry and Biology (IFM), Linköping University, S-581 83 Linköping, Sweden*

[2] *ITMO University, Kronverkskiy pr. 49, 197101 St. Petersburg, Russian Federation*

[3] *St-Petersburg Academic University Khlopina 8/3, 194021 St. Petersburg, Russian Federation*

[4] *Ioffe Institute, Politekhnicheskaya 26, 194021 St. Petersburg, Russian Federation*

[5] *Department of Physics, Durham University, South Road, Durham, UK DH1 3LE*

* galia.pozina@liu.se

Photoluminescence

Low-temperature photoluminescence (PL) spectra for the Bragg MQW structure taken from the sample top are shown in Fig. S1 for different delay times after the laser pulse (the spectra are shown within ~800 ps total interval). The emission angle was fixed at 40° and the excitation power was 27 mW. The time interval between spectra in Fig. S1 is ~43 ps. The feature at 1.47 eV corresponds to the X1 exciton state in InAs QW, while the broader line at ~1.48 eV at the bottom spectrum is related to the super-radiant (SR) mode with peak position shifting to lower energies with increasing delay time. The position of the X1 line is constant. There is a change of the relative intensity between the X1 line and the SR mode with increasing delay time: while the X1 line intensity increases within shown period, there is an obvious decrease of the SR emission intensity.

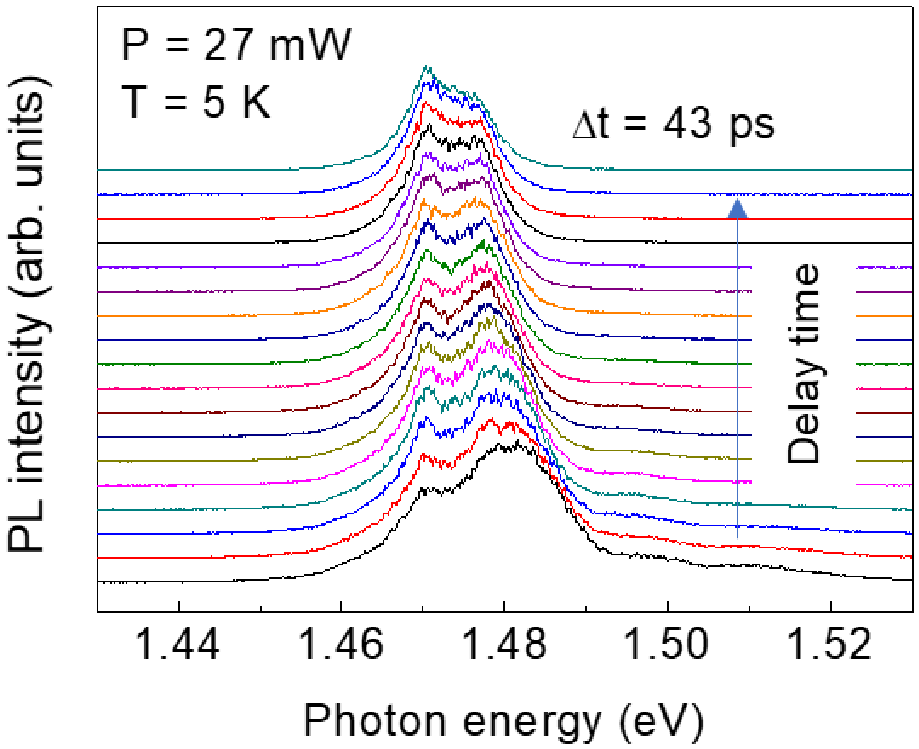

Figure S1. PL spectra at 5 K are shown for different delay times. Spectra are shifted vertically for clarity. The emission angle is 40°.

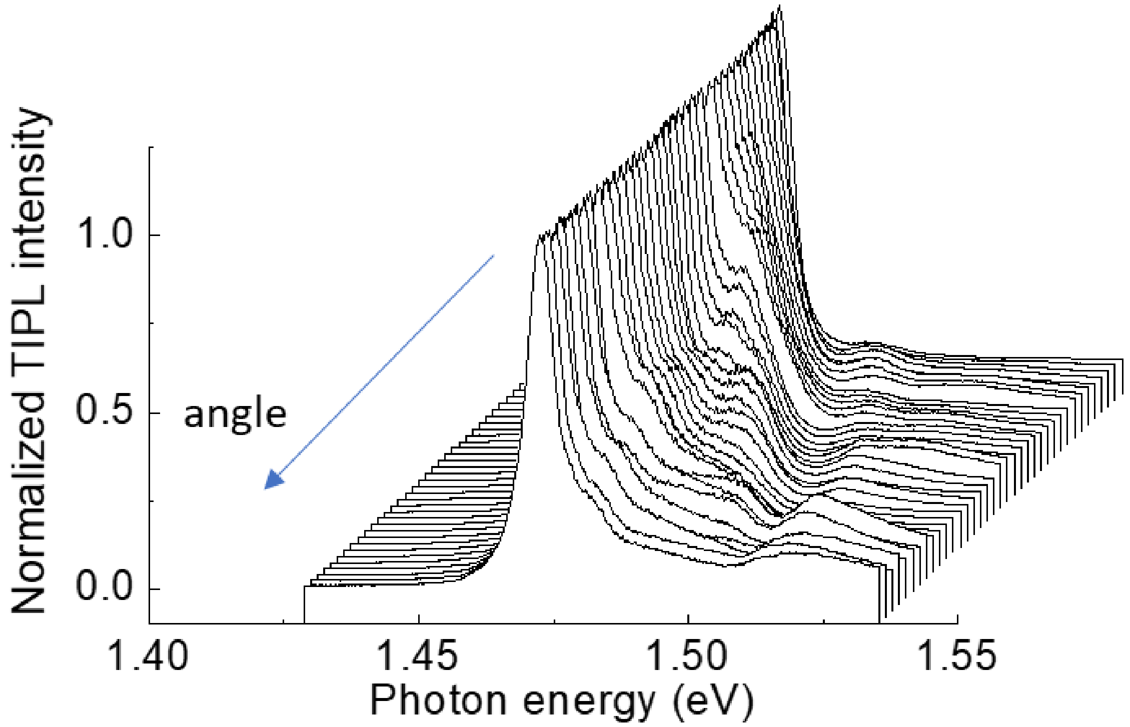

Figure S2. TI-PL spectra measured at temperature of 5 K and at the excitation power of 27 mW for the Bragg MQW structure at different emission angles from 15° to 88° with a step of 2°.

We have measured time-integrated PL (TI-PL) and time-resolved PL (TRPL) data for the emission angles between 15° and 88°. Measurements at the same conditions for angles below 15° was limited by the experimental setup. The normalized TI-PL spectra are plotted in Fig. S2 for different angles. The behaviour of the SR mode (high energy shoulder) shows some peculiar (non-monotonic) behaviour vs angle. Such behaviour is more apparent in TRPL images measured at the same angles. Examples of TRPL images are shown for angles from 36° to 44° in Fig. S3 (top). The bottom of Fig. S3 shows corresponding TI-PL spectra. In TI-PL spectra the SR mode is not resolved, while in TRPL both the X1 line and SR modes are well-resolved and show different temporal behaviour.

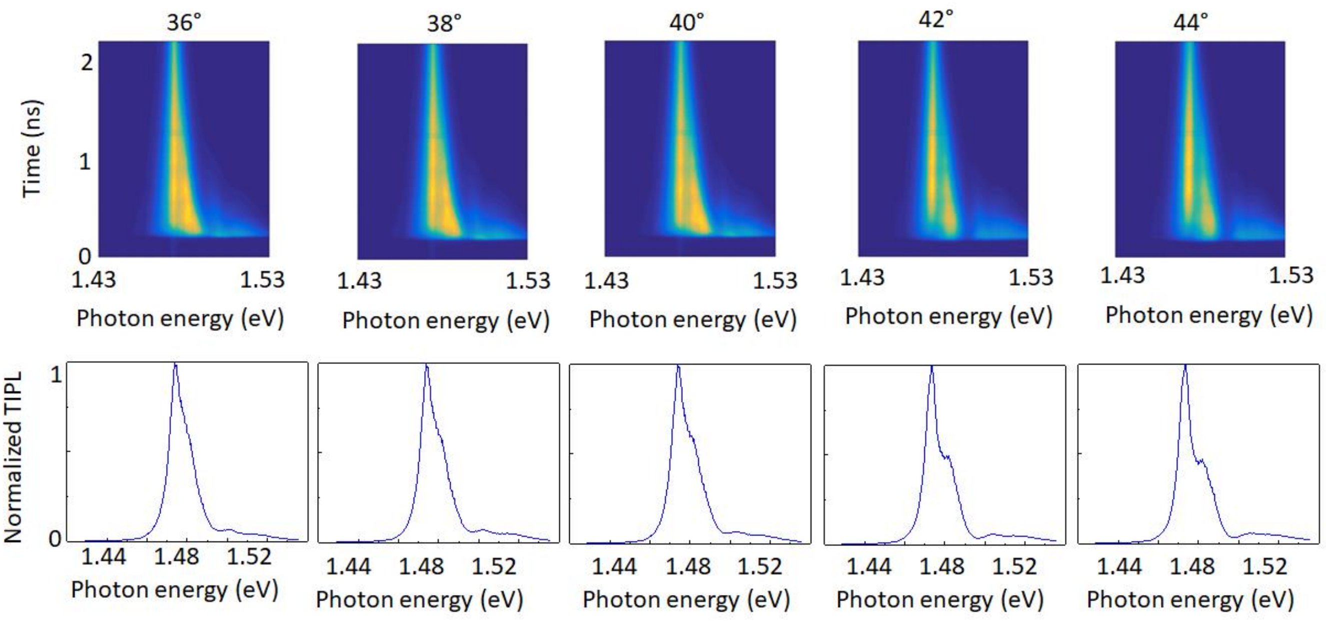

Figure S3. Top - TRPL images taken for the Bragg MQW structure at 5 K at different emission angles. Excitation power is 27 mW for all spectra. Bottom – corresponding TI-PL spectra measured at the same conditions and angles.

Calculation of reflection coefficients

The reflection coefficient as seen in Fig. 3 of the main text was calculated using the transfer matrix method in the basis of longitudinal (tangential) components of the electrical

and magnetic field. We will assume that the structure is surrounded by the media of the refractive index n_0 to the left and n_1 to the right. It is helpful to choose the coordinates in such a way that the z axis is perpendicular to the structure layers and for the light wavevector $k_y = 0$ and thus $k_x = \cos\theta\, n_0\omega/c$ is conserved during the crossing of the interfaces. In the case of TE polarization the basis vector is $[E_y, H_x]$, and in the case of TM polarization it is $[H_y, E_x]$. A transfer matrix for a uniform non-magnetic layer of thickness d and refractive index n (which can be a function of the frequency ω) has the form

$$M(n,d) = \begin{pmatrix} \cos k_z d & -\frac{i}{p}\sin k_z d \\ -ip\sin k_z d & \cos k_z d \end{pmatrix} \qquad \text{(S1)}$$

where

$$k_z = \sqrt{n^2 k_0^2 - k_x^2} \qquad \text{(S2)}$$

$$p = k_z/k_0 \qquad \text{(S3)}$$

for TE polarization and

$$p = k_z/(n^2 k_0) \qquad \text{(S4)}$$

for TM polarization. The transfer matrix for the leap across the interface in this basis is unity.

The QW layer has a different transfer matrix dependent on its reflection coefficient

$$r_{QW} = \frac{i\Gamma}{\omega_0 - \omega - i(\Gamma + \gamma)} \qquad \text{(S5)}$$

Here ω_0 is the exciton resonance frequency, Γ and γ are radiative and non-radiative decay rates. The transfer matrix has the form

$$M_{QW} = \begin{pmatrix} 1 & 0 \\ -2p\frac{r}{1+r} & 1 \end{pmatrix} \qquad \text{(S6)}$$

for TE-polarized light and

$$M_{QW} = \begin{pmatrix} 1 & -2p\frac{r}{1+r} \\ 0 & 1 \end{pmatrix} \qquad \text{(S7)}$$

for TM-polarized light. The value of p is calculated according to the medium enclosing the QW.

The full structure is described by the transfer matrix M which is the product of the matrices for the individual layers. The reflection and transmission coefficients are given by:

$$r = \frac{(M_{11}+M_{12}p_1)p_0-(M_{21}+M_{22}p_1)}{(M_{11}+M_{12}p_1)p_0+(M_{21}+M_{22}p_1)} \quad \text{(S8)}$$

$$t = \frac{2p_1}{(M_{11}+M_{12}p_1)p_0+(M_{21}+M_{22}p_1)} \quad \text{(S9)}$$

Here the values of $p_{0,1}$ are calculated just as in equation (S3-S4) with $n = n_{0,1}$.

The values of the parameters used for calculation of reflection coefficients are summarized in Table S1.

Calculation of the Purcell factor

We presented here a detailed description of the S-quantisation procedure used to obtain results shown in Fig. 6 of the main text. The numerical values used are presented in Table S1, and the reflection and transmission coefficients discussed below are calculated as described above in Section “Calculation of reflection coefficients”.

Solution of a wave equation for electromagnetic field in infinite uniform media with refractive index n

$$\boldsymbol{\nabla} \times \boldsymbol{\nabla} \times \boldsymbol{E} = n^2 \left(\frac{\omega}{c}\right)^2 \boldsymbol{E} \quad \text{(S10)}$$

gives a continuous spectrum of eigenfrequencies of the mode ω. In order to provide a quantum-mechanical description of the interaction of radiation and matter, the field should be quantized: continuous spectrum of EM modes should be replaced by a discrete one. For this purpose, EM field is considered in a “quantization box” of “large” size (see Fig. S4) and

boundary conditions (BC) are to be set on the facets of the box[1]. The natural choice is to set periodic Born-Karman BC:

$$\begin{cases} E|_{x=0} = E|_{x=L_x} \\ \left.\frac{\partial E}{\partial x}\right|_{x=0} = \left.\frac{\partial E}{\partial x}\right|_{x=L_x} \end{cases} \quad \text{(S11)}$$

$$\begin{cases} E|_{y=0} = E|_{x=L_y} \\ \left.\frac{\partial E}{\partial y}\right|_{y=0} = \left.\frac{\partial E}{\partial y}\right|_{y=L_y} \end{cases} \quad \text{(S12)}$$

$$\begin{cases} E|_{z=0} = E|_{z=L_z} \\ \left.\frac{\partial E}{\partial z}\right|_{z=0} = \left.\frac{\partial E}{\partial z}\right|_{z=z} \end{cases} \quad \text{(S13)}$$

Wave equations (S10) with BC equations (S11-S13) can be considered as eigenvalue and eigenfunction problems and the solution of the problem is given by a discrete set of wavevectors $\boldsymbol{k} = (k_x, k_y, k_z)$ obeying

$$k_{x,y,z} = \pm 2\pi n N_{x,y,z}/L_{x,y,z}, \quad \text{(S14)}$$

where $N_{x,y,z}$ are integers; corresponding eigenfunctions have the form of propagating planewaves:

$$E = E_0 \exp\left(i(k_x x + k_y y + k_z z)\right) \quad \text{(S15)}$$

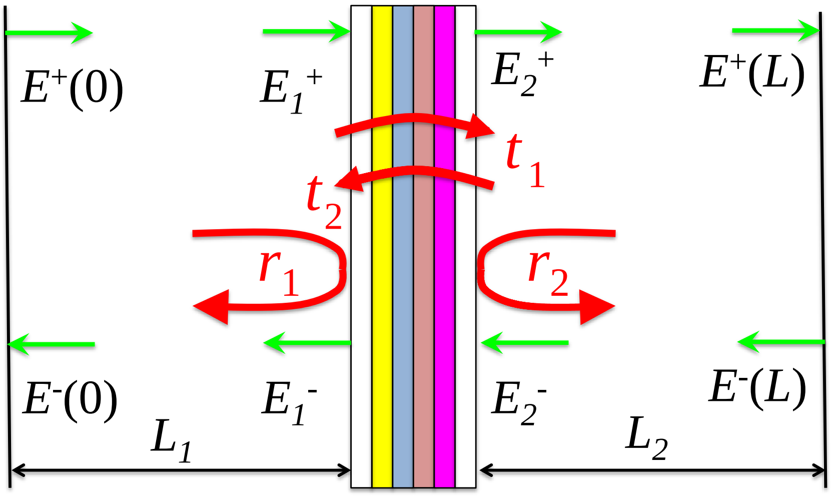

Figure S4. Inhomogeneous structure in the quantization box.

We should note that the same set of eigenvalues of the wave vector is provided by equating eigenvalues of the transfer matrix $\widehat{M}$ along each direction x, y, and z through the quantization box to unity.

An analysis provided above leads to an expression for the density of states in K-space:

$$\rho_k = \frac{dN}{dk_x dk_y dk_z} = \frac{n^3 V}{(2\pi)^3} \tag{S16}$$

where $V = L_x L_y L_z$ is the volume of quantization box. Then, an expression for the density of states in respect to the frequency (energy) reads as

$$\rho = \frac{dN}{d(\hbar\omega)} = \frac{dN}{dK}\frac{dK}{d(\hbar\omega)} = \frac{dN}{dK}\frac{dK}{d(\hbar\omega)} = \frac{n^3\omega^2}{\pi^2 c^3}\frac{V}{\hbar} \tag{S17}$$

Each EM mode can be considered as a quantum oscillator with an energy of $\hbar\omega/2$, and this energy should be associated with an integral of the density of EM energy of the mode over quantization box[2]:

$$\frac{1}{4\pi}\int_V n^2 E^2 d^3\boldsymbol{r} = \hbar\omega/2. \tag{S18}$$

Equation (S16) and (S18) allow to obtain Fermi golden rule in the form which does not depend on a virtual quantization box[3]:

$$W = \alpha |\langle f|\boldsymbol{\epsilon r}|i\rangle|^2 \frac{4n\omega^3}{c^2} \ , \tag{S19}$$

here we introduce dimensionless function $\boldsymbol{\epsilon}$ describing spatial distribution of the electric field of the mode satisfying relation $\boldsymbol{E} = E_0\boldsymbol{\epsilon}$, where $\alpha = e^2/(\hbar c) \approx 1/137$ is the fine structure constant and $\boldsymbol{\epsilon}$ is a normalized vector describing an electric field of the EM mode. Note that function $\boldsymbol{\epsilon}$ satisfies the normalization condition

$$\frac{1}{4\pi}\int_V \epsilon^2 d^3\boldsymbol{r} = 1 \tag{S20}$$

As was noted above, the periodic BC can be set by equating eigenvalues of the transfer matrix through a uniform quantization box to unity, providing a set of eigenvalues in the form of wavevectors. When inhomogeneity is inserted into the quantum box, then wavevectors are

not good quantum numbers anymore[4,5]. On the other hand, adequate description of inhomogeneous structure can be given by a scattering matrix, which couples the waves incident on the structure (incoming waves) and outgoing waves.

We propose the procedure of quantization of the electromagnetic field, based on equating to unity eigenvalues of scattering matrix of the system, or by equating incoming amplitudes and outgoing amplitudes.

Now we will define the quantization procedure in detail. Let us consider a quantization box with a layered structure within, as shown in Fig. S4. The distances from the left and the right facet of the quantization box to layered structures are L_1 and L_2. In the case of such layered structure, it is convenient to consider mode of electromagnetic field with specific angular frequency ω in the form:

$$E_{K_x,K_y}(x,y,z) = E(z)\exp(iK_x\, x)\exp\left(iK_y\, y\right) \tag{S21}$$

where the lateral components of the wavevector K_x and K_y relate to the direction of propagation of the waves in empty parts of the quantization box via relations:

$$K_x = \frac{\omega}{c}\sin\theta\cos\varphi \tag{S22}$$

and

$$K_y = \frac{\omega}{c}\sin\theta\sin\varphi. \tag{S23}$$

In the case of TE polarization electric field of the wave has the component E_y only, while for TM polarization there are components E_x and E_z.

In each layer of the structure, spatial dependence of the electric field along the z-axis is defined as the superposition of the waves propagating in opposite directions along the z-axis, and in the subsequent discussion we denote the wave with positive K_z with upper index "$+$", for negative K_z we will use upper index "$-$".

We denote amplitudes of the waves incident on the left and right facets of the quantization box as $E^{+}(0)$ and $E^{-}(L)$, and amplitudes of the waves outgoing from right and left boundaries as $E^{+}(L)$ and $E^{-}(0)$.

Amplitudes of the waves on left and right facets of the quantization box are coupled by relation

$$\begin{pmatrix} \lambda_2^* E_{K_x,K_y}^{+}(L) \\ \lambda_1^* E_{K_x,K_y}^{-}(0) \end{pmatrix} = \begin{pmatrix} t_1 & r_1 \\ r_2 & t_2 \end{pmatrix} \begin{pmatrix} \lambda_1 E_{K_x,K_y}^{+}(0) \\ \lambda_2 E_{K_x,K_y}^{-}(L) \end{pmatrix} \tag{S24}$$

where r_1 and r_2 are the amplitude reflection coefficients of layered structure for the waves incident from the left and right sides respectively, t_1 and t_2 are the corresponding amplitudes of the transmission coefficient of the layered structure, and the phases gained by waves propagating from the facets of quantization boxes to layered structures are given by $\lambda_{1,2} = \exp(iK_z L_{1,2})$. It follows that amplitudes of incoming waves $\left[E_{K_x,K_y}^{+}(0),\ \ E_{K_x,K_y}^{-}(L)\right]$ are coupled with amplitudes of outgoing waves $\left[E_{K_x,K_y}^{+}(L),\ \ E_{K_x,K_y}^{-}(0)\right]$ by scattering matrix $\hat{S}$

$$\begin{pmatrix} E_{K_x,K_y}^{+}(L) \\ E_{K_x,K_y}^{-}(0) \end{pmatrix} = \hat{S} \begin{pmatrix} E_{K_x,K_y}^{+}(0) \\ E_{K_x,K_y}^{-}(L) \end{pmatrix} \tag{S25}$$

and $\hat{S}$ reads as

$$\hat{S} = \begin{pmatrix} \lambda_1\lambda_2 t_1 & \lambda_2^2 r_2 \\ \lambda_1^2 r_1 & \lambda_1\lambda_2 t_2 \end{pmatrix}. \tag{S26}$$

Eigenvalues of $\hat{S}$ matrix are:

$$\beta_{1,2} = \lambda_1\lambda_2 \left(\frac{t_1+t_2}{2} \pm \sqrt{\left(\frac{t_1-t_2}{2}\right)^2 + r_1 r_2} \right) \tag{S27}$$

and related eigenvectors are:

$$B^{(1,2)} = \left[1, -\frac{\lambda_1}{\lambda_2} \left(\frac{t_2-t_1}{2r_2} \pm \sqrt{\left(\frac{t_2-t_1}{2r_2}\right)^2 + \frac{r_1}{r_2}} \right) \right] \tag{S28}$$

In the important case of a non-absorbing system or for any system possessing center of symmetry eigenvalues have a simpler form:

$$\beta^{(1,2)} = \lambda_1\lambda_2(t \pm \sqrt{r_1 r_2}), \qquad \text{(S29)}$$

and the eigen-vectors are:

$$\mathrm{B}^{(1,2)} = \left[1, \quad \pm(\lambda_1/\lambda_2)\sqrt{\mathrm{r}_1/\mathrm{r}_2}\right]. \qquad \text{(S30)}$$

It is clear that for a symmetric quantization box (when $L_1 = L_2$) the eigen-vectors depend only on the properties of the inhomogeneity, but not on the size of quantization box.

We then proceed by equating the eigenvalues of the scattering matrix $\hat{\mathrm{S}}$ to unity:

$$\beta^{(1,2)} = 1 \qquad \text{(S31)}$$

Solution of equation (S31) in respect to frequency thus gives the spectrum of eigenfrequencies. Using the set of quantum numbers, one can obtain the eigenvectors $B^{(1)}$ and $B^{(2)}$, and calculate the field profile of the mode using the transfer matrix method. The components of the eigenvectors $B^{(1,2)}$ are the complex amplitudes of the fields incident on the edges of the box, and the field of the mode is the superposition of the fields, excited by waves incident on the structure from opposite directions, and corresponding spatial profiles of the electric field described by the functions $\tilde{\boldsymbol{\epsilon}}^{(1,2)}$.

Similar to the case of periodic BC, we can consider the mode obtained using S-quantization as an elementary quantum oscillator and normalize it using equation (S18). The field of the mode should be normalized according to equation (S20). We denote BC, equation (S31), as S–conditions, and the procedure of quantization described above as S-quantization.

If the size of the quantization box goes to infinity, then the contribution of the layered structure to value of integral (equation (S18)) will be negligible, and the integral equation (S18) will be equal to the contribution given by the wave in empty parts of quantization box. Thus, the amplitude of electric field of EM mode, normalized using equation (S18) incident

on empty quantization box, and incident on quantization box with layered structure, will be equal.

Since density of the states provided by S-quantization is the same density of states as the setting periodic BC, for the specific EM mode probability of spontaneous emission given by equation (S19) for the dipole in layered structure will be defined by modification of the amplitude of field vector $\tilde{\boldsymbol{\epsilon}}$ in the layered structure. This modification (spatial profile of the field within the layered structure) does not depend on the size of the left and right empty parts of quantization box and is defined only by reflection coefficients r_1 and r_2 and transmission coefficients t_1 and t_2 of the layered structures. Thus, the size of the empty part of a quantization box can be reduced to zero.

An approach based on modification of the spatial profile of the modes in microcavities has been used by De Martini[6], though the use of the periodic BC limits an applicability of the results obtained in this work. An approach used there corresponds to the use of only "symmetric" eigenvector $B^{(1)}$, while the mode corresponding to "antisymmetric" eigenvector $B^{(2)}$ is missed. This is only viable if an emitter is placed at the centre of a symmetric structure, since the value of the mode field corresponding to $B^{(2)}$ is zero in the centre of a symmetric structure. If the dipole is placed in an arbitrary place in a structure without specific symmetry, the modes corresponding to both $B^{(1)}$ and $B^{(2)}$ should be taken into account.

For a development of the formalism, it is convenient to relate components of vector $\tilde{\boldsymbol{\epsilon}}^{(1,2)}$ describing electric field in layered structure to the components of vector $\boldsymbol{\epsilon}^{(1,2)}$ for uniform medium via coefficients X, Y and Z as specified below. For TE mode:

$$\tilde{\boldsymbol{\epsilon}}^{(1,2)} = \begin{pmatrix} 0 & \tilde{\epsilon}_y^{(1,2)} & 0 \end{pmatrix} = (0 \quad Y^{(1,2)}\epsilon_y^{(1,2)} \quad 0) \qquad \text{(S32)}$$

while for TM mode:

$$\tilde{\boldsymbol{\epsilon}}^{(1,2)} = \begin{pmatrix} \tilde{\epsilon}_x^{(1,2)} & 0 & \tilde{\epsilon}_z^{(1,2)} \end{pmatrix} = (X^{(1,2)}\epsilon_x^{(1,2)} \quad 0 \quad Z^{(1,2)}\epsilon_z^{(1,2)}) \qquad \text{(S33)}$$

It is also convenient to define the *modal Purcell factor* for a specific mode characterized by direction of propagation defined by the polar angle θ of wave in free space as a ratio of probability of spontaneous emission for this mode to probability of spontaneous emission in the free space, when dipole is parallel to the field of the mode:

$$F_{\theta}^{(TE)} = \frac{|\langle f|\tilde{\boldsymbol{\epsilon}}\boldsymbol{r}|i\rangle|^2}{|\langle f|\epsilon r|i\rangle|^2} \tag{S34}$$

Such definition of the modal Purcell factor will be convenient for the subsequent analysis of the Purcell effect in the case of waveguide modes.

The dot product in the equation (S34) for TE modes reads as

$$\tilde{\boldsymbol{\epsilon}}^{(1,2)}\boldsymbol{r} = \tilde{\epsilon}_y^{(1,2)} r_y = Y^{(1,2)}\epsilon_y^{(1,2)} r_y \tag{S35}$$

and for the TM mode:

$$\tilde{\boldsymbol{\epsilon}}^{(1,2)}\boldsymbol{r} = \tilde{\epsilon}_x^{(1,2)} r_x + \tilde{\epsilon}_z^{(1,2)} r_z = X^{(1,2)}\epsilon_x^{(1,2)} r_x + Z^{(1,2)}\epsilon_z^{(1,2)} r_z \tag{S36}$$

Therefore, the Purcell factor for the specific TE mode characterized by the emission angle θ is

$$F_{\theta}^{(TE)} = \sum_{i=1,2}\left|Y^{(i)}\right|^2 \left(r_y/r\right)^2 = \sum_{i=1,2}\left|Y^{(i)}\right|^2 \sin^2\varphi_{\boldsymbol{d}} \sin^2\theta_{\boldsymbol{d}} \tag{S37}$$

while for TM mode:

$$F_{\theta}^{(TM)} = \sum_{i=1,2}\left|X^{(i)}\frac{\epsilon_x^{(i)}}{|\epsilon^{(i)}|}\frac{r_x}{r} + Z^{(i)}\frac{\epsilon_z^{(i)}}{|\epsilon^{(i)}|}\frac{r_z}{r}\right|^2 =$$

$$= \sum_{i=1,2}\left|X^{(i)}\cos\theta\cos\varphi_{\boldsymbol{d}}\sin\theta_{\boldsymbol{d}} + Z^{(i)}\sin\theta\cos\theta_{\boldsymbol{d}}\right|^2 \tag{S38}$$

In the case of TE polarization, for the dipole oriented along y-axis, the modal Purcell factor is just

$$F_{\theta}^{(TE)} = \sum_{i=1,2}\left|Y^{(i)}\right|^2 \tag{S39}$$

For the dipole oriented along axis *Ox*, the Purcell factor for TM modes is

$$F_{\theta}^{(TM)} = \sum_{i=1,2}\left|X^{(i)}\right|^2 \cos^2\theta \tag{S40}$$

and for the orientation of dipole along *Oz* axis:

$$F_{\theta}^{(TM)} = \sum_{i=1,2} \left| Z^{(i)} \right|^2 \sin^2 \theta \qquad \text{(S41)}$$

Thus, the quantities X, Y, and Z define the probability of spontaneous emission in layered structure within the light cone.

Since the size of the quantization box does not influence the components of eigenvectors $B^{(1,2)}$ we can exclude the quantization box from consideration. Thus, for the construction of eigen-vectors and the functions $\tilde{\boldsymbol{\epsilon}}^{(1,2)}$, one can take the values of the reflection and transmission coefficients of inhomogeneity at its interfaces.

References

1 Mandel, L. & Wolf, E. *Optical coherence and quantum optics*. (1995).

2 Coccioli, R., Boroditsky, M., Yablonovitch, E., Rahmat-Samii, Y. & Kim, K. W. Smallest possible electromagnetic mode volume in a dielectric cavity. *IEE Proceedings - Optoelectronics* **145**, 391-397, doi:10.1049/ip-opt:19982468 (1998).

3 Bykov, V. P. Spontaneous emission in a periodic structure. *Soviet Journal of Experimental and Theoretical Physics* **35**, 269 (1972).

4 Kaliteevski, M., Mazlin, V., Ivanov, K. & Gubaydullin, A. Quantization of electromagnetic field in an inhomogeneous medium based on scattering matrix formalism (S-quantization). *Optics and Spectroscopy* **119**, 832-837 (2015).

5 Kaliteevski, M. A., Gubaydullin, A. R., Ivanov, K. A. & Mazlin, V. A. Quantization of electromagnetic field and analysis of Purcell effect based on formalism of scattering matrix. *Optics and Spectroscopy* **3**, 410-419 (2016).

6 De Martini, F., Marrocco, M., Mataloni, P., Crescentini, L. & Loudon, R. Spontaneous emission in the optical microscopic cavity. *Physical Review A* **43**, 2480 (1991).

Table S1. Values of the parameters used for calculation.

n_0	1
n_1& n (bulk)	3.5
ω_0 (X1)	1.4725 eV
ω_0 (X2)	1.4825 eV
ω_0 (X3)	1.4925 eV
Γ (X1)	0.0350 meV
Γ (X2)	0.0175 meV
Γ (X3)	0.0135 meV
γ (X1)	0.6 meV
γ (X2)	0.6 meV
γ (X3)	0.6 meV
